# Zinc Supplementation Associated With a Decrease in Mortality in COVID-19 Patients: A Meta-Analysis

**DOI:** 10.7759/cureus.40231

**Published:** 2023-06-10

**Authors:** Spencer Z Rheingold, Chirag Raval, Antonio M Gordon, Patrick Hardigan

**Affiliations:** 1 Research, Dr. Kiran C. Patel College of Allopathic Medicine, Davie, USA; 2 Internal Medicine, University Health Care, Hialeah, USA

**Keywords:** covid and zinc, covid 19 mortality, zinc, supplementation, sars-cov-2, coronavirus, sars cov 2, zinc supplementation, covid 19, covid

## Abstract

The COVID-19 pandemic has had a significant impact on the world, resulting in millions of deaths worldwide and imposing economic, political, and social problems. The use of nutritional supplementation for the prevention and mitigation of COVID-19 remains controversial. This meta-analysis aims to investigate the association between zinc supplementation, mortality, and symptomatology, among COVID-19-infected patients. A meta-analysis was conducted to compare the outcomes of mortality and symptomology of patients with COVID-19 receiving zinc supplementation and those not receiving zinc supplementation. PubMed/Medline, Cochrane, Web of Science, and CINAHL Complete were independently searched with the search terms "zinc" AND "covid" OR "sars-cov-2" "COVID-19" OR "coronavirus". After duplicates were removed, 1215 articles were identified. Five of these studies were used to assess mortality outcomes, and two were used to assess symptomatology outcomes. The meta-analysis was conducted through R 4.2.1 software (R Foundation, Vienna, Austria). Heterogeneity was evaluated by calculating the I^2^ index. The Preferred Reporting Items for Systematic Reviews and Meta-Analyses (PRISMA) guidelines were used. It was found that COVID-19-infected individuals treated with zinc supplements had a reduced risk of mortality compared with individuals not treated with a zinc supplement RR=0.63 (95%CI;0.52,0.77), p=0.005. For symptomology, it was found that COVID-19-infected individuals treated with zinc had no difference in symptomology than individuals not treated with a zinc supplement RR=0.52 (95%CI;0.00,24315.42), p=0.578. This data indicates that zinc supplementation is associated with decreased mortality in those with COVID-19 but does not change symptomatology. This is promising as zinc is widely available and may be valuable as a cost-effective way to prevent poor outcomes for those with COVID-19.

## Introduction and background

The first reports of the SARS-COV-2 virus that came out of Wuhan, China, in 2019, which led to the COVID-19 pandemic, is still being spread across the world today. The widespread administration of COVID-19 vaccines and the restrictive measures put in place worldwide have led to restrictions being removed and mitigation of excessive spreading of the virus and poor outcomes. According to the World Health Organization (WHO), as of March 21st, 2023, there have been 761,071,826 confirmed cases worldwide and 6,879,677 confirmed deaths due to the SARS-COV-2 worldwide [1].

The first reports of the novel coronavirus infection presented with pneumonia. It is now known that COVID-19 symptoms can range from asymptomatic or mild, with symptoms of fever, fatigue, chills, sore throat, cough, loss of smell, and loss of taste. COVID-19 can also lead to severe disease due to an overactive immune system that results in excessive release of cytokines, increased oxidative stress, and activation of pro-coagulation factors [2]. This can lead to acute respiratory distress syndrome, multi-organ failure, and death [3]. Certain co-morbidities such as hypertension, diabetes, chronic obstructive pulmonary disease (COPD), obesity, and cardiovascular diseases have been shown to increase the risk of severe disease and death [1].

There have been immense efforts across many platforms, such as the Centers for Disease Control and Prevention (CDC), WHO, and private companies to find effective treatment and prevention against COVID-19. The CDC and WHO have prioritized resources to focus on the creation or testing of diagnostic tools, immunologic therapies, anti-viral drugs, and vaccine candidates [1]. There is limited supportive data on the effectiveness of nutritional supplementation in the prevention and outcome mitigation of the COVID-19 virus infection. Many research and clinical groups have conducted randomized-control trials and case-control studies to investigate the effectiveness of zinc as nutritional supplementation for mitigating poor outcomes in COVID-19 patients [4].

The second most abundant trace element is zinc, one of the most important in humans [5]. The amount of total zinc in the human body is two to four grams, with a plasma concentration of 12-16 μM [6]. According to the National Academies of Sciences, the recommended daily allowance is 11 mg/day and 8 mg/day of zinc for adult males and females, respectively [7]. Zinc is vital for the function and growth of all cells. Specifically, it has been found that zinc is an important modulator of the immune system in the setting of inflammation and infection. Zinc is involved in the innate and adaptive immune system, increasing polymorphonuclear cell and macrophage chemotaxis and phagocytosis. Zinc is involved in oxidative-guided pathogen killing [8-9]. Zinc also regulates the proliferation, maturation, and differentiation of lymphocytes [10]. Zinc deficiency has been shown to increase the release of proinflammatory cytokines, such as interleukins IL-1β, IL-6, and tumor necrosis factor (TNF)-α [11].

Zinc also has direct anti-viral properties. Zinc has been recognized as therapeutic against other upper respiratory pathogens, such as the common cold [12-14]. It has been shown that increased intracellular zinc concentrations results in decreased SARS-COV-2 replication [15-17]. The angiotensin-converting enzyme (ACE) receptor that is used by SARS-COV-2 to gain entry into host cells is regulated by zinc, possibly decreasing its expression [18,19]. Zinc has also been shown to increase the viral entry of medications such as chloroquine and hydroxychloroquine, decreasing viral replication [20,21].

This review aims to give insight into the existing literature on the efficacy of zinc supplementation as a means of poor-outcome mitigation in SARS-COV-2 virus-infected individuals.

## Review

Methods

This meta-analysis was carried out in accordance with the Preferred Reporting Items for Systematic Reviews and Meta-Analyses (PRISMA) guidelines. 

Search strategy

**Figure 1:**
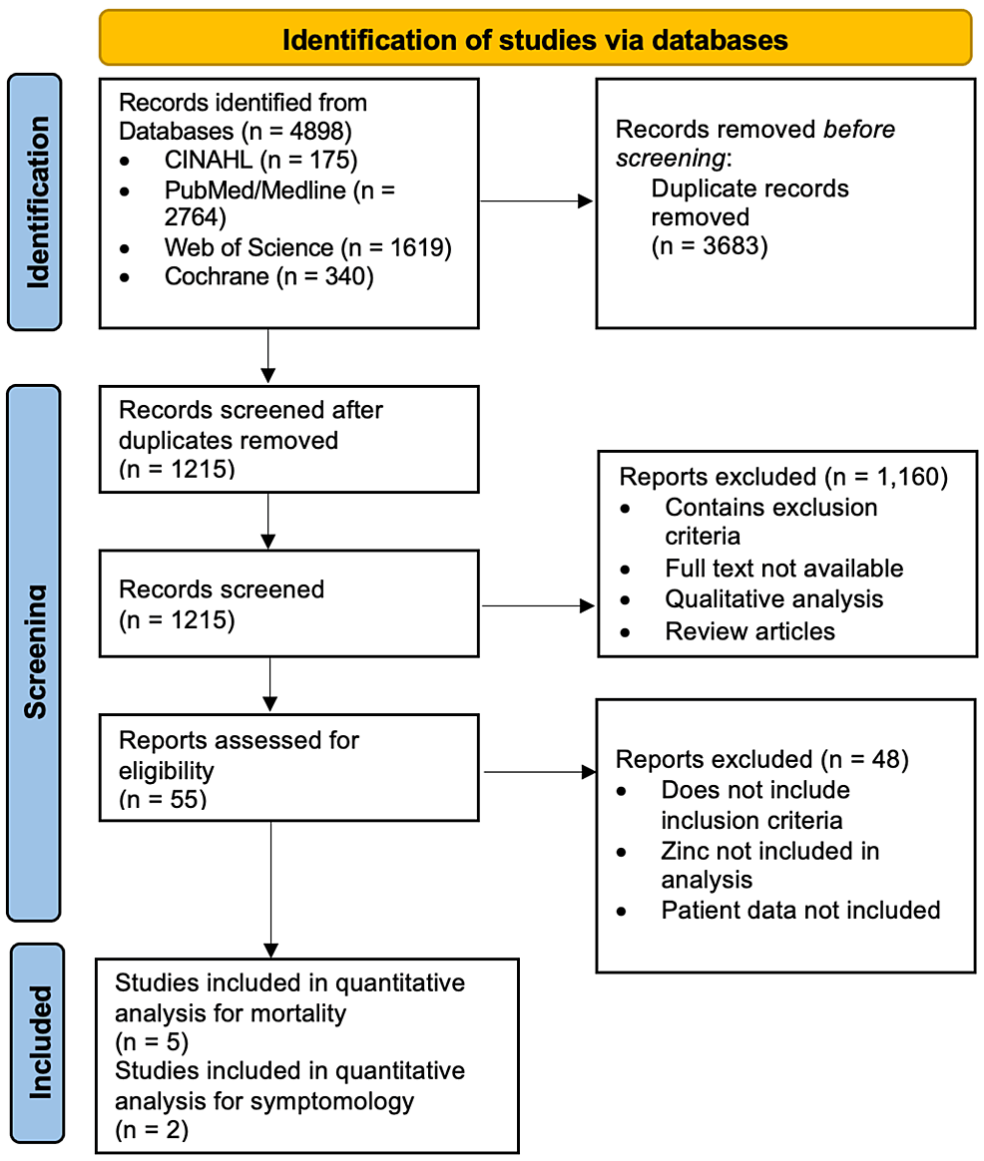
Search strategy flowchart for meta-analysis literature search

This meta-analysis was constructed from a search by two authors, Spencer Rheingold (SR) and Chirag Raval (CR), between July 2022 and August 2022. PubMed/Medline, Cochrane, Web of Science, and CINAHL Complete were independently searched. For each database, an initial search was conducted using the following search terms: "zinc" AND "covid" OR "sars-cov-2" "COVID-19" OR "coronavirus". Duplicates were then removed from the initial search. From there, studies that contained the exclusion criteria described below were excluded from the study. Lastly, studies that did not include the inclusion criteria were removed, which lead to the studies used in this paper. Figure 1 demonstrates the search strategy.

Inclusion and exclusion criteria

Using the Population, Exposure/Intervention, Comparison/Control, and Outcome (PECO/PICO) strategy, the studies that met the inclusion and exclusion criteria were included in the study. The inclusion criteria were based on three parameters: the populations studied, the exposure/intervention used, and the study outcomes. The study must have had subjects that were assessed on the impact of zinc supplementation on COVID-19 infection. There must have been a subject group that was supplemented with zinc and a control group that did not receive zinc. The studies must have assessed the outcomes of mortality and/or asymptomatic/mild symptomatology and severe symptomatology. Two subject groups were required when selecting papers that assessed symptomology, one with less severe COVID-19 infection and another with severe COVID-19 infection. If there was more than one group, the least severe and the most severe group were chosen for analysis. The exclusion criteria consisted of studies with no accessible full-text, studies that did not report specific outcomes quantitatively, and no abstracts, comments, reviews, posters, and editorial reviews.

Study selection

Two authors (SR and CR) screened each paper independently, looking at the titles and abstracts for possible eligibility. The studies were further evaluated, looking over the full text and determining if the exclusion and inclusion criteria were met. The final selection of papers was reviewed by a third author (Patrick Hardigan (PH)). Figure 1 shows the breakdown of how the final papers were selected.

Risk of bias assessment

The risk of bias was assessed on a consensus three-point Likert scale (high, some concerns, and low) using the following criteria: bias due to randomization, bias due to deviations from intended interventions, bias due to missing data, bias due to outcome measurement, bias due to selection of reported result. No papers were included or excluded based on these criteria.

Statistical analysis

The meta-analysis was conducted through R 4.2.1 software (R Foundation, Vienna, Austria). Heterogeneity was evaluated by calculating the I^2^ index. I^2^ values less than 25%, 25-50%, 50-75%, and 75-100% were homogeneous or had low, medium, and high heterogeneity levels, respectively. The random effect model (REM) was applied if the I^2^ value was > 50%, while the fixed effect model (FEM) was applied if the I^2^ value was <50%. The combined risk ratio (RR) with corresponding 95% confidence intervals (CI) was used to assess the relationship between supplements and mortality, supplements and symptoms among COVID-19-infected individuals. Both mortality and symptomology were operationalized as yes/no variables. The mortality and symptomatology data for each study are shown in Tables 1 and 2, respectively.

**Table 1:** Data collected from studies that assessed mortality outcomes on zinc supplementation

Author	Year	Study	Treatment, N	Treatment success	Treatment failure	Control, N	Control success	Control failure
Al Sulaiman et al. [22]	2021	Retrospective cohort	82	340	42	82	19	63
Carlucci et al. [23]	2020	Retrospective cohort	411	257	54	402	402	119
Gordon et al. [24]	2021	Randomized-control	104	104	0	95	95	1
Thomas et al. [25]	2021	Randomized-control	87.9	87.9	0	88	88	0
Abd-Elsalam et al. [26]	2021	Retrospective cohort	96	91	5	90	90	5
Total			780			882		

**Table 2:** Data collected from studies that assessed symptomatology outcomes on zinc supplementation

Author	Year	Study	Treatment, N	Asymptomatic/mild	Symptomatic	Control, N	Asymptomatic/mild	Symptomatic
Gordon et al. [24]	2021	Randomized-control	104	102	2	96	86	10
Abd-Elsalam et al. [26]	2021	Retrospective cohort	96	9	87	95	12	83
Total			200			691		

Results

**Figure 2:**
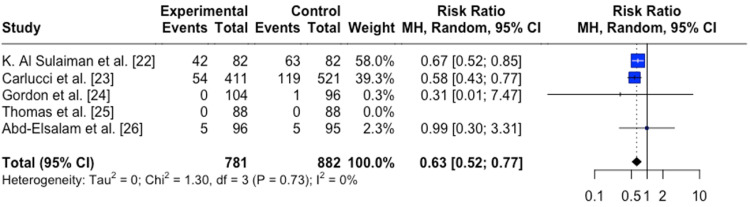
Forest plot for the risk difference of mortality for COVID-19 infected patients between patients treated with zinc vs. control patients not treated with zinc

For mortality, it was found that COVID-19-infected individuals treated with zinc supplement had a reduced risk of mortality than individuals not treated with a zinc supplement RR=0.63 (95%CI;0.52,0.77), p=0.005 (Figure 2). In this analysis, two studies demonstrated a significant association between zinc supplements and mortality, whereas three others failed to reach significance. The two studies reaching statistical significance were retrospective observational studies [22,23].

**Figure 3:**
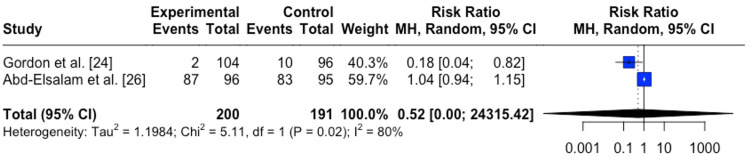
Forest plot for the risk difference in symptomology for COVID-19 infected patients between patients treated with zinc vs. control patients not treated with zinc

**Figure 4:**
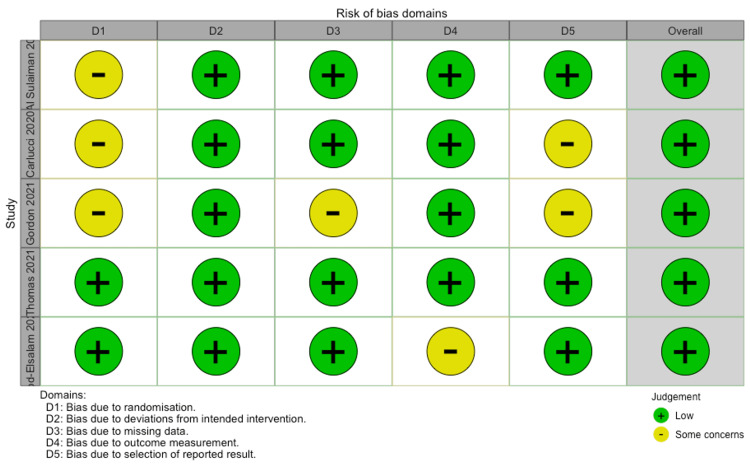
Risk of bias traffic light plot

For symptomatology, it was found that COVID-19-infected individuals treated with zinc supplement had no difference in symptomology than individuals not treated with a zinc supplement RR=0.52 (95%CI;0.00,24315.42), p=0.578 (Figure 3). Both studies used in this analysis were prospective randomized trial studies [24,26]. For both analyses (mortality and symptomology) risk of bias was low (Figure 4).

Discussion

The data concluded that zinc supplementation was associated with a decrease in mortality in those with COVID-19 but has no impact on symptomatology. This data is promising as zinc is widely available and may be used as a cost-effective way to prevent poor outcomes for those with COVID-19. This data also opens up the possibility of further research on the effectiveness of zinc against other viral infections.

The mechanism behind zinc's action in the body supports this data. Many of the deaths due to COVID-19 are due to septic shock, acute respiratory distress syndrome (ARDS), and multi-organ failure [27]. Adequate zinc levels have been shown to decrease the release of pro-inflammatory cytokines, such as interleukins IL-1β, IL-6, and tumor necrosis factor (TNF)-α, which can contribute to the over-responsive immune system leading to ARDS, septic shock and multi-organ failure [11,27]. Also, zinc's activity in immune cell proliferation and activity, inhibition of SARS-COV-2 replication, and decrease in ACE expression can all lead to the prevention of severe infection and severe outcomes that lead to death [15,18,19].

There has been no other meta-analysis that has assessed zinc supplementation and COVID-19 mortality. A Tabatabaeizabeh study concluded a decrease in mortality with zinc supplementation, while the Szarpak et al. study concluded no significant decrease in risk of mortality with zinc supplementation [28,29]. These papers did not assess symptomology between experimental and control groups. Another meta-analysis was done by Hunter et al. that investigated 28 randomized control trials with zinc supplementation, but these were not specific to SARS-COV-2. They found that zinc supplementation was associated with decreased severity and duration of symptoms [30].

There were limitations to this study. A small number of studies were assessed, only five were chosen for mortality analysis with a total sample size of 1,474 patients, and two were chosen for symptomology with a sample size of 391 patients. The design of the studies chosen limits the conclusions made in this study regarding mortality as three of the studies are retrospective studies and only two are randomized control trials for mortality. Both papers that assessed symptomology were randomized control trials. Between studies, zinc formulations were different, and in some cases, zinc was given in combination with other drugs.

## Conclusions

This meta-analysis showed an association between zinc supplementation and a reduction in mortality in COVID-19 patients but no evidence for its effect on decreasing symptomology of the COVID-19 infection. With the possible benefits displayed in this meta-analysis, along with its cost-effectiveness and availability, zinc may be a viable approach to reducing mortality in COVID-19 patients. More research on zinc supplementation as a supportive treatment or prophylaxis of the COVID-19 virus before any conclusions can be made on its effectiveness.
